# Translating the immediate effects of *S*-Ketamine using hippocampal subfield analysis in healthy subjects-results of a randomized controlled trial

**DOI:** 10.1038/s41398-021-01318-6

**Published:** 2021-04-01

**Authors:** Anna Höflich, Christoph Kraus, Ruth M. Pfeiffer, Rene Seiger, Dan Rujescu, Carlos A. Zarate, Siegfried Kasper, Dietmar Winkler, Rupert Lanzenberger

**Affiliations:** 1 Department of Psychiatry and Psychotherapy, Medical University of Vienna, Vienna, Austria; 2 Experimental Therapeutics and Pathophysiology Branch, National Institute of Mental Health, National Institutes of Health, Bethesda, MD USA; 3 Biostatistics Branch, National Cancer Institute, National Institutes of Health, Bethesda, MD USA; 4 Department of Psychiatry, Psychotherapy and Psychosomatics, Martin-Luther-University Halle-Wittenberg, Halle, Germany

**Keywords:** Molecular neuroscience, Depression

## Abstract

Antidepressant doses of ketamine rapidly facilitate synaptic plasticity and modify neuronal function within prefrontal and hippocampal circuits. However, most studies have demonstrated these effects in animal models and translational studies in humans are scarce. A recent animal study showed that ketamine restored dendritic spines in the hippocampal CA1 region within 1 h of administration. To translate these results to humans, this randomized, double-blind, placebo-controlled, crossover magnetic resonance imaging (MRI) study assessed ketamine’s rapid neuroplastic effects on hippocampal subfield measurements in healthy volunteers. S-Ketamine vs. placebo data were analyzed, and data were also grouped by brain-derived neurotrophic factor (*BDNF*) genotype. Linear mixed models showed that overall hippocampal subfield volumes were significantly larger (*p* = 0.009) post ketamine than post placebo (LS means difference=0.008, standard error=0.003). Post-hoc tests did not attribute effects to specific subfields (all *p* > 0.05). Trend-wise volumetric increases were observed within the left hippocampal CA1 region (*p* = 0.076), and trend-wise volumetric reductions were obtained in the right hippocampal—amygdaloid transition region (HATA) (*p* = 0.067). Neither genotype nor a genotype–drug interaction significantly affected the results (all *p* > 0.7). The study provides evidence that ketamine has short-term effects on hippocampal subfield volumes in humans. The results translate previous findings from animal models of depression showing that ketamine has pro-neuroplastic effects on hippocampal structures and underscore the importance of the hippocampus as a key region in ketamine’s mechanism of action.

## Introduction

Ketamine has emerged as a potent and rapid-acting treatment for patients with difficult-to-treat depression and acute suicidal ideation, leading to a major interest in its mechanism of action^1,2^. Ketamine’s rapid clinical antidepressant effects begin about 60-120 minutes after the start of the infusion and peak 24 h post administration; at this timepoint, ketamine itself is fully eliminated and only metabolites such as dehydronorketamine and hydroxynorketamine (HNK) are detectable^3^. Ketamine is a non competitive NMDA receptor antagonist first introduced in clinics as a dissociative anesthetic. The molecular mechanisms underlying its antidepressant properties involve a number of targets, including the glutamatergic and monoaminergic neurotransmitter systems, γ-aminobutyric acid (GABA), opioid and cholinergic receptors, among others^4,5^.

One of its several major mechanisms of antidepressant action is the restoration of synaptic plasticity deficits, which is in line with evidence of decreased dendrites in animal models of depression as well as in depressed patients^6^. In animals, mechanistic studies found that ketamine triggered the release of brain-derived neurotrophic factor (BDNF)/TrkB, with downstream effects on the mechanistic target of rapamycin (mTOR) via α-amino-3-hydroxy-5-methyl-4-isoxazolepropionic acid receptors (AMPARs) and L-type voltage-dependent Ca^2+^channels^7^. Concordantly, ketamine’s antidepressant effects were found to depend on adequate functioning of AMPARs as well as BDNF and mTOR^6,8,9^. However, differences in the cellular mechanisms of antidepressant action have been reported between the *R*- and *S*-ketamine enantiomers, particularly with regard to mTORC1 activation^10^. Furthermore, NMDA receptor blockade by ketamine has been reported to deactivate eukaryotic elongation factor-2 (eEF2 kinase), thus reducing eEF2 phosphorylation and increasing BDNF translation^11^. Genetic knockout or blockage of *BDNF* leads to impaired synaptic remodeling and prevents antidepressant-like response to ketamine in medial prefrontal cortical neurons^12^.

In addition, in vitro studies with hippocampal pyramidal neurons found immediately enhanced pyramidal cell excitability mediated by reduced synaptic inhibitory input after ketamine administration^13^. At the cellular level, ketamine restored stress-induced synaptic deficits in the prefrontal cortex (PFC) in mice 24 h post infusion^14^. Ketamine also restored apical dendritic spine deficits in CA1 pyramidal neurons 1 h after infusion^15^, a timepoint that coincides with the onset of anti-depressant-like effects in Flinders Sensitive Line (FSL) rats^16^. Taken together, these results suggest that ketamine rapidly facilitates synaptic plasticity and neuronal function within pre frontal and hippocampal circuits.

With regard to *BDNF*, the Met allele of a single-nucleotide polymorphism (SNP), Val66Met, has been linked to impaired activity-dependent BDNF secretion in in-vitro experiments^17^ and associated with reduced hippocampal volumes in healthy individuals^18^ and depressed patients^19^, although null findings have also been reported^20^. Met carriers have also shown less robust anti-depressant response to ketamine compared to Val carriers^21^. Ketamine-induced synaptogenesis was found to be impaired in Met/Met knock-in mice, with failure to increase spine density and spine head diameter in layer V pyramidal cells of the PFC^22^. In addition, the *BDNF* Val66Met genotype modulates the anti-depressant-like effects of the (*2* *R,6* *R)*-HNK metabolite in rodents^23^. However, to date, no translational study has examined the relationship between ketamine’s neuroplastic mechanisms and *BDNF* genotype status in humans.

This study examined whether the immediate neuroplastic effects of ketamine seen in animal models were observable in human subjects receiving *S*-ketamine. Data from a placebo-controlled pharmacological magnetic resonance imaging (MRI) ketamine study were used to examine hippocampal substructures measured with sequential structural MRIs (sMRIs) and hippocampal subfield measurements^24^. Notably, changes in automatically calculated sMRI volumes have been linked to microscopically validated synaptic spine density^25^. The design of the present study corresponded to a recent study by Treccani et al.^15^ that reported rapid increases in dendritic spine density in hippocampal CA1 pyramidal neurons using *S*-ketamine in an animal model of depression. It was hypothesized that human subjects would exhibit increased CA1 regional volume after ketamine infusion versus placebo. As a secondary hypothesis, ketamine-induced volumetric changes associated with carrying the *BDNF* Val66Met Met-allele were also examined.

## Materials and methods

### Subjects

All procedures were approved by the Ethics Committee of the Medical University of Vienna. The study was registered with the European Union Drug Regulating Authorities Clinical Trials (EudraCT Nr. 2010-022772-31) and approved by the Austrian Federal Office for Safety in Health Care. The study was also registered on clinicaltrials.gov (NCT01394757). Details regarding the design and results of this study that focused on other research questions have previously been published^24,26–28^.

Briefly, this analysis included data from 31 healthy subjects (14 females, mean age: 25.2) who participated in a double-blind, randomized, placebo-controlled, cross-over sMRI study in 2011–2012. The sample size was similar to comparable MRI studies in this field^29,30^. Written informed consent was obtained from all participants included in the study. Prior to MRI scans, physical and mental health was assessed based on medical examination, a psychiatric interview, the Structured Clinical Interview for DSM-IV (SCID), electrocardiogram, and blood and urine analyses, including a pregnancy and drug test. Individuals were excluded if they had a current psychiatric or physical illness, a first-degree relative suffering from a major psychiatric disorder, or lifetime history of any relevant psychiatric, neurological, or somatic disorder, current or former substance abuse, treatment with psychotropic agents within the last 6 months, or a lifetime use of antipsychotic drugs. All individuals with contraindications against MRI were also excluded. Random allocation sequence was provided by the Center for Medical Statistics, Informatics and Intelligent Systems, Medical University of Vienna. Randomization (block randomization) and provision of the blinded study medication were performed by the hospital pharmacy at the General Hospital of Vienna. Sealed envelopes with the information about the drug were provided to the study team together with the study medication for any case of emergency. These envelopes were stored in a locked deposit until the end of the study, when the unblinding for all participants was provided by the pharmacy. The study team and the participants were blinded to the order of medication until the end of the study.

### Structural MRI and hippocampal subfield segmentation

**Fig. 1:**
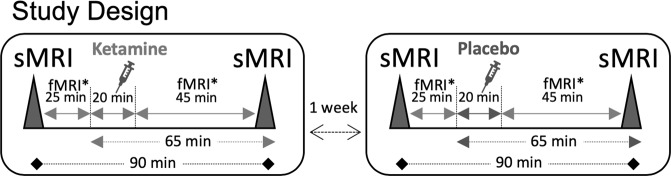
Study design. Thirty-one healthy subjects (mean age: 25.2 years) underwent two magnetic resonance imaging (MRI) scans—one with ketamine, the other with placebo (saline)—in a randomized order at least 1 week apart. Each subject received a bolus of 0.11 mg/kg *S-*ketamine hydrochloride followed by a maintenance infusion of 0.12 mg/kg over 20 min. Structural MRI (sMRI) sequences were obtained at baseline and 65 min after the start of the infusion. As part of an earlier study, each subject underwent functional MRI (fMRI) before and after sMRI.

All included subjects underwent two scanning sessions at 3 Tesla each, including two structural T1-weighted MRIs (Siemens Trio, Erlangen, Germany) under both placebo and ketamine conditions. At both study visits, each subject had a baseline sMRI before and a second sMRI after drug administration within the same scanning session without being repositioned. The second sMRI scan started 65 min after the start of the *S*-ketamine or placebo infusion (Fig. 1). At a second study visit that was scheduled between 7 and 14 days after the first visit, all subjects underwent a scan with the crossover drug and identical scanning parameters. *S*-ketamine was given as a bolus of 0.11 mg/kg, followed by a maintenance infusion of 0.12 mg/kg over a period of 20 min. Placebo consisted of saline using the same flow velocity as *S*-ketamine. The mean total dosage of ketamine was 15.49 ± 2.9 mg.

sMRI was conducted with turboflash-3D MPRAGE sequences with repetition time (TR)/echo time (TE)=2300/4.21 ms, a field of view of 240×256 mm with 160 sagittal slices and flip angle set to 9°, and a final voxel size of 1.0×1.0×1.1 mm. Neuropsychological scales (the Positive and Negative Syndrome Scale (PANSS) and the Five-Dimensional Altered States of Consciousness Scale (5D-ASC)) were conducted at a desk outside the scanner room immediately before and after MRI scanning. As previously reported, no significant difference in movement within the scanner was observed between the ketamine and placebo conditions during functional MRI (fMRI) scans performed immediately after the sMRI scans as part of a prior study^24^.

The segmentation of hippocampal subfields was done using FreeSurfer image analysis suite v6.0 beta-version (Athinoula A. Martinos Center for Biomedical Imaging, Charlestown, MA, http://surfer.nmr.mgh.harvard.edu/) with the longitudinal processing option^31^. FreeSurfer was recently reported to have good test-retest reliability for the segmentation of hippocampal subfields^32^. The following subfields were defined (in alphabetical order): CA1, CA3, CA4, fimbria, fissure, granule cell layer of the dentate gyrus, hippocampus–amygdala transition area, molecular layer, parasubiculum, presubiculum, subiculum, and tail. Whole-brain segmentation for calculating the total intracranial volume (TIV) was performed in SPM12 (Wellcome Centre for Human Neuroimaging, London, UK, https://www.fil.ion.ucl.ac.uk/spm/) and using Matlab 8.3 scripts (The MathWorks, Inc. Natick, MA). Quality control of subfield segmentation was performed visually after segmentation and showed no misalignments in the sample.

### Single nucleotide polymorphism genotyping of BDNF

The assay for SNP rs6265 (*BDNF* Val66Met) was performed as previously described^33^. First, 9 ml of blood from each subject was collected in EDTA blood tubes. DNA isolation was performed using the QiaAmp DNA blood maxi kit (Qiagen, Hilden, Germany); genotyping was done using the iPLEX assay on the MassARRAY MALDI-TOF mass spectrometer. Selection of allele-specific extension products and genotype assignment were performed using Typer 3.4 Software (Sequenom, San Diego, CA, USA). Quality criteria (individual call rate >80%, SNP call rate >99%, and identification of genotyped CEU trios (Coriell Institute for Medical research, Camden, NJ) with HapMap database >99%) were applied and met.

### Statistical analysis

First, the influence of ketamine or placebo on total hippocampal volume (THV) was calculated in a preliminary analysis in order to assess potential global hippocampal volumetric changes. Volumetric THV differences were calculated in each hemisphere before and after ketamine or placebo administration and compared with paired two-sample Student’s *t*-tests.

To investigate the ultra-rapid effects of ketamine on hippocampal subfields, linear mixed models were fit as follows. First, the absolute volumetric differences between each sMRI sequence (post-pre ketamine administration) were calculated for each condition, and the resulting values were log-transformed to yield the dependent variable *Y*_*ijkl*_ for subject *i*, drug *j*, regimen *k* (specifically, order of drug or placebo) and region *l.* The linear mixed effects model (LMM) *Y*_*ijkl*_ = *μ* + *α*_j_ + *γ*_*k*_ + *αγ*_*jk*_ + *δ*_*l(i)*_+*β*_*j(l)*_+*X*_*i*_ + *ε*_*ijkl*_ was then fit, where *μ* is overall mean log volumetric differences, *α*_*j*_ is the (fixed) effect of drug *j, γ*_*k*_ is the (fixed) effect for regimen *k* (specifically, order of drug or placebo), *αγ*_*jk*_ is an interaction term for drug with regimen, *δ*_*l(i)*_ is the random effect for a specific region *l* nested within person *i*, *β*_*i(k*)_ is the random effect for nesting of subjects within measures, and *ε*_*ijkl*_ is the error, assumed to be independent and random. Models were adjusted for covariates *X*, including sex, age, and TIV. The influence of *BDNF* Val66Met genotype status on hippocampal subfield changes was tested by incorporating genotype and genotype *x* drug interaction in the linear mixed model. As in previous neuroimaging genetics studies, Met/Met (*n* =2) and Val/Met (*n* =10) allele carriers were combined into one group and compared to subjects carrying Val/Val (*n* = 19)^21^. Because a previous study suggested that THV provides a more accurate measure^34^, LMMs were also adjusted for THV instead of TIV in sensitivity analyses. Least squares means and standard errors (SEs) were computed. In each model, post-hoc tests of ketamine versus placebo were also calculated and corrected for multiple comparisons with Tukey’s method as implemented in SAS PROC MIXED, with an alpha of *p* < 0.05. All analyses were conducted in SAS 9.4 (SAS Institute Inc., Cary, NC, USA).

## Results

As assessed by PANSS total score, PANSS subscales, and 5D-ASC scores, *S*-ketamine infusion led to robust clinical effects (all *p*-values ≤ 0.001).

The LMM adjusted for sex, age, and TIV showed that ketamine led to significantly larger hippocampal subfield volumes than placebo at 65 min post infusion (difference of least squares means: 0.008, SE = 0.003, *p* = 0.009). Whether subjects received ketamine or placebo first in the crossover design had no significant effect, nor was there any significant interaction between drug and regimen (*F*_1,742_ = 0.01, *p* = 0.93), suggesting that there were no carryover effects for the participants who received ketamine for the first sMRI scan. The results were generally similar when the model was adjusted for THV instead of TIV, with a slightly stronger main effect observed for drug (*p* = 0.008).

When the effects of *BDNF* Val66Met genotype were examined as a factor with potential influence on hippocampal subfield dynamics under ketamine versus placebo conditions, no significant main effect of genotype (*F*_1,742_ = 6.79, *p* = 0.75) was observed in a model that included Val/Val versus pooled Val/Met and Met/Met genotypes (19 Val/Val; 12 Met carriers). Similarly, no drug x *BDNF* genotype interaction was noted (*F*_1,742_ = 0.09, *p* = 0.77).

**Fig. 2:**
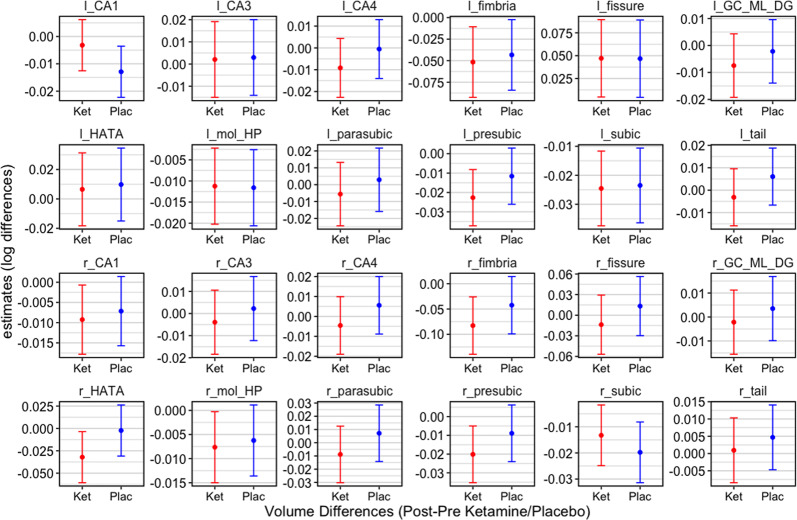
Mixed model estimates of ketamine versus placebo in hippocampal subfields. Linear mixed effects modelling of estimates of volumetric differences 65 min post-ketamine and post-placebo administration. Trend-wise differences between ketamine and placebo were observed in the left CA1 (*p* = 0.076) and the right HATA (*p* = 0.067). Bars represent estimated means and 95% confidence intervals adjusted for sex, age, and total hippocampal volume. Values correspond to those in Table 1. CA1, CA2, CA3, of the ammon’s horn; GC-ML-DG, granule cells, molecular layer, dentate gyrus; HATA, hippocampal-amygdaloid transition area; mol_HP, molecular layer hippocampus.

Post-hoc analyses comparing each subfield’s volumetric differences found no significant differences between ketamine and placebo conditions (all corrected *p* > 0.05; Table 1). However, volumetric differences with trend-wise significance were observed between ketamine and placebo for the right hippocampal-amygdaloid transition region (mean difference = −0.030, SE = 0.016, corrected *p* = 0.067) and the left CA1 (mean difference=0.010, SE = 0.005, corrected *p* = 0.076; Fig. 2).

**Table 1:** Post-hoc results of volumetric differences in the ketamine versus placebo condition for all included subfields.

Region	Estimate	Adjusted Lower	Adjusted Upper	Adjusted p-values
Overall	0.008	0.001	0.014	**0.009**
CA1_l	0.010	−0.020	0.001	**0.076**
CA1_r	−0.002	−0.0070	0.011	0.634
CA3_l	−0.001	−0.023	0.025	0.939
CA3_r	−0.006	−0.014	0.027	0.542
CA4_l	−0.009	−0.010	0.028	0.361
CA4_r	−0.010	−0.010	0.030	0.318
GC_ML_DG_l	−0.005	−0.011	0.022	0.520
GC_ML_DG_r	−0.006	−0.013	0.024	0.545
HATA_l	−0.003	−0.030	0.037	0.844
HATA_r	−0.030	−0.002	0.062	**0.067**
Fimbria_l	−0.008	−0.049	0.066	0.770
Fimbria_r	−0.040	−0.039	0.12	0.310
Fissure_l	0.000	−0.053	0.052	0.988
Fissure_r	−0.027	−0.020	0.075	0.255
mol_HP_l	0.000	−0.012	0.011	0.947
mol_HP_r	−0.001	−0.009	0.011	0.777
Parasubic_l	−0.008	−0.018	0.035	0.517
Parasubic_r	−0.020	−0.008	0.040	0.178
Presubic_l	−0.011	−0.003	0.025	0.117
Presubic_r	−0.011	−0.007	0.030	0.227
Subic_l	−0.001	−0.017	0.019	0.907
Subic_r	0.006	−0.023	0.010	0.423
Tail_l	−0.009	−0,009	0.027	0.302
Tail_r	−0.004	−0.009	0.017	0.567

Results corrected for multiple comparisons with Tukey’s method. CA1, CA2, CA3, of the ammon’s horn; GC-ML-DG, granule cells, molecular layer, dentate gyrus; HATA, hippocampal–amygdaloid transition area; mol_HP, molecular layer hippocampus; overall: overall hippocampal subfields. Results are further visualized in Fig. 2.

**Fig. 3:**
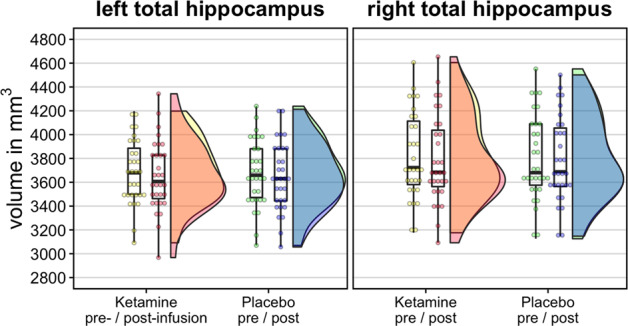
Absolute volumes of the left and right hippocampi before and after ketamine and placebo administration. A paired *t*-test comparison of absolute volumetric differences before and after ketamine or placebo found that neither ketamine nor placebo had any effect on the total hippocampal volume (all *p* > 0.05).

Finally, no significant difference in THV changes for each hemisphere was observed between ketamine and placebo (left hippocampus: mean difference between ketamine and placebo = −8.25mm^3^, t_30_ = −0.62, *p* = 0.61; right hippocampus: mean difference = −16.2 mm^3^, t_30_ = −0.93, *p* = 0.34; Fig. 3).

## Discussion

This study found that, in healthy human subjects, *S*-ketamine had a significant effect on overall hippocampal subfield volume 65 minutes post infusion compared to placebo. To our knowledge, this study is the first to use sMRI to address this topic in humans, confirming a finding that has been repeatedly reported in animal studies that used direct measures to quantify neuroplastic changes. Furthermore, this effect could not be attributed to specific subfields. Rather, significantly larger volumes were observed over all subfields post ketamine compared to post placebo administration, with the highest—although only of trend-wise significance—increases observed in the right CA1 region. The *BDNF* Val66Met genotype was not significantly associated with hippocampal subfield dynamics. Taken together, the results add to pre-existing evidence from animal studies of ketamine’s rapid neuroplastic effects in the hippocampus and extend our knowledge of ketamine’s mechanism of action in humans.

Interestingly, current theories of the pathophysiology of depression as well as the mechanism of action of antidepressants in general, and ketamine in particular, support the present finding that ketamine affects overall hippocampal subfield volumes^8^. For instance, sMRI studies found that hippocampal volume was decreased in depressed patients^35–38^. Significant decreases in total hippocampal volume, particularly in the CA1 subregion, have been also reported in animals using the chronic unpredictable mild stress model of depression^39,40^. In addition, antidepressant treatment has been shown to increase neurogenesis in the hippocampus of rats^41^ as well as increase synaptic protein levels and dendritic outgrowth in the hippocampal neurons of rats^42^. Increased hippocampal volume has also been observed in depressed patients after monoaminergic antidepressant treatment and electroconvulsive therapy (ECT)^43,44^, although null results have also been reported^45^. It should be noted here that a previous study found significantly more pronounced clinical effects post ketamine administration in depressed patients with smaller hippocampal volumes prior to treatment, at 24 h post infusion; however, this study did not investigate the direct effects of ketamine on hippocampal volume^29^.

This study sought to translate the results obtained by Treccani and colleagues, who demonstrated that ketamine restored synaptic deficits in the CA1 region of the hippocampus within 60 min of administration in the FSL rat model of depression^15^. The present study thus aimed to record ketamine’s direct, short-term effects in a way that matched the study design of that animal study. The question of the most favorable timepoint for detecting ketamine-induced effects on neuroimaging parameters with maximum effects and specificity has not been clearly answered. Some authors argue that the strongest effects are evident up to 24 hours after ketamine administration, which may be particularly true for clinical effects. However, previous animal and functional neuroimaging studies by our group and others found that ketamine’s effects were apparent within a short time period^9,15,24,26,46^. Notably, in the study by Treccani and colleagues, only animals in the FSL model exhibited this rapid increase in spine density, and the effect was absent in Flinders Resistant Line control animals^15^. However, ketamine’s synaptogenic effects have also been observed in unstressed, wild-type animals^9^.

The dose of *S*-ketamine used in this study was based on both previous investigations and a pilot study conducted to establish the dose needed to achieve robust clinical effects, while the maximizing tolerability of the MRI measurement in order to obtain good data quality. With regard to comparability with animal studies, no clear evidence has yet been extrapolated regarding the ketamine dosage administered intraperitoneally in animals versus intravenously in humans. However, a mouse study by Highland and colleagues found that intravenous (2 *R*,6*R*)-HNK infusion led to higher cerebral bioavailability than intraperitoneal administration^47^. Another study by Le Nedelec and colleagues reported approximately 15-fold higher peak plasma concentrations for the *R*- and *S*-enantiomers of ketamine using intravenous bolus administration compared to subcutaneous, intramuscular, or continuous intravenous infusion, but the study did not examine intraperitoneal administration^48^.

Given the lack of methods to directly measure synaptogenesis in humans in vivo, indirect measures such as high- resolution sMRI or positron emission tomography (PET) with radio ligands binding at synaptic vesicles such as [^11^C]UCB-J predominate the field^49^. As a result, mass changes in synapses influence sMRI signals, including change in the number of dendrites or synapses^50^. One study elegantly demonstrated that stress-induced gray matter reductions in mice were related to dendritic spine density loss in the CA1 and to dendritic length reductions in the hippocampus, thus confirming that sMRI can be successfully used to indirectly measure neuroplasticity^51^. In addition, Autry and colleagues found a significant increase in BDNF protein levels 30 min post ketamine infusion in mice, supporting the notion that rapid plasticity changes are a neurobiological substrate of rapidly changing hippocampal subfield volumes^46^. Recently, Moda-Sava and colleagues identified links between loss of dendritic spines in an animal model of chronic stress and disruption of microcircuits within the PFC, both of which were restored by ketamine^14^. Ketamine has also been found to restore connectivity between the ventral hippocampus and the medial PFC, effects that can be mimicked by high- frequency stimulation of the hippocampus^52^. These findings further strengthen the notion that ketamine’s rapid synaptogenic effects lead to rapid changes in hippocampal structures, thereby affecting the connections of glutamatergic projections into the PFC^53^. However, sMRI signals also underlie non-neuronal components, which could provide an alternative explanation for our results. In that regard, altered brain vasculature or water content, microglial changes, and changes caused by the MRI procedure itself, such as scanner drift or head motion, might be responsible for volumetric changes. However, as noted above, no significant differences were observed regarding movement during the MRI scan between the ketamine and placebo conditions, as assessed via the fMRI scans performed immediately after the sMRI scans.

Finally, this study assessed the impact of the Val66Met *BDNF* genotype based on previous reports of differences in ketamine-induced synaptogenesis in animals^22^, BDNF turnover, and dendritic spine density in the CA1 region in animals^54^, and differences in antidepressant response between genotypes^21^. However, in the present study, Val66Met *BDNF* genotype status (Val/Val vs. Met carriers) was not associated with hippocampal subfield volumetric changes in response to either ketamine or placebo. This lack of significant results in the present analysis could either reflect low power due to the limited sample size or that the polymorphism may have a weaker effect in healthy volunteers compared to depressed patients.

### Limitations

Several limitations need to be considered when interpreting our results. First, hippocampal spatial resolution and tissue contrast could have been further optimized by applying higher field strengths such as 7 T and concurrent recording of T2-weighted images, as others have previously suggested^55^. Second, the *BDNF* genotype analysis had limited power due to the study’s small sample size, which was not able to detect interactions. Third, some studies have suggested that the T1-signal may be susceptible to non specific influences such as changes in blood flow. However, the placebo-controlled study design, the association between synaptic spine density and results of studies using voxel-based morphometry, and the fact that our results reflect preclinical evidence in this field of research support the present results^25^. Fourth, further temporal development of the measured effects remains beyond the scope of our study, so the persistence of these changes and their importance for sustained ketamine effects need to be investigated in future studies.

It is also important to stress that, because this study was performed in humans, a number of technical differences exist between this study and the animal study published by Treccani and colleagues^15^. In particular, because Treccani and colleagues did not include an MRI scan to directly correlate the effect of dendritic remodeling with volumetric changes, it is difficult to directly compare the results. Evidence of a direct association between dendritic arborization and hippocampal volumes in animal models of depression is missing; thus, conclusions drawn from any comparisons between the current study and that of Treccani and colleagues, as well as other animal studies, remain indirect, although strongly supported by the current literature.

Finally, another possible limitation of this study is the relatively low dose of ketamine used. As noted above, this dose was used based on the results of an earlier pilot study (*n*=10). Of note, this dose led to significant clinical effects. Animal studies using relatively low doses of ketamine have also observed significant effects on spine density^56^. However, translational studies that use higher ketamine dosages are warranted to replicate our results.

## Conclusion

In summary, these results provide the first evidence that ketamine has rapid effects on hippocampal subfield volumes in humans. The findings echo those observed in previous animal models of depression that ketamine’s rapid effects on hippocampal morphology might contribute to its antidepressant effects. Future studies are needed to further investigate optimum ketamine dosage and scanning timepoints and to replicate these findings in depressed patients.
